# In Praise of Wisdom: A Morality Tale

**DOI:** 10.1089/jpm.2022.0325

**Published:** 2022-09-30

**Authors:** Harvey Max Chochinov

**Affiliations:** ^1^ Department of Psychiatry, University of Manitoba, Winnipeg, Manitoba, Canada.; ^2^ CancerCare Manitoba Research Institute, Winnipeg, Manitoba, Canada.

**Keywords:** bioethics, health care policy, medical assistance in dying, morality, palliative care smart, wisdom

## Abstract

Wisdom and intelligence work best in unison. What happens, however, when seemingly smart people fail to exercise wisdom, either in social discourse, clinical encounters, or even within the broader political arena? This morality tale, in which Wisdom and Smart take each other on in a debate at a local bar, illustrates the fallout, when these two are not on the same page.

## Introducing the Cast

Medicine is fraught with controversy, particularly when engaging issues wherein life and death hang in the balance. In those instances, being smart allows one to sift through the complexity of information that may have a bearing on how things will play out. But intelligence alone is often insufficient. Lack of wisdom may see us arrive at solutions that are arguably right, but do not feel right; or can be justified, but do not feel just. With your indulgence, I have asked *Smart* and *Widsom* to take part in this morality tale. I suspect they will explain this far better than I' can.

Smart talks. Wisdom listens. Smart tends to be loud, whereas Wisdom is soft spoken. Smart always has answers. Wisdom tries hard to understand the questions. Smart moves quickly, whereas Wisdom takes its time. Smart can be bold and flashy. Wisdom is more subdued and humble. Smart always has something to say, whereas Wisdom knows that silence is sometimes the best response.

There is something incredibly attractive, even seductive, about Smart. Smart thinks quickly on its feet, always has a comeback and can nail a sound bite. Smart plays well on social media; Wisdom, not so much. Smart draws attention to itself and makes convincing pronouncements. Listening to Smart, things seem straightforward and black or white. People looking for simple solutions to difficult questions—in social situations, clinical encounters, or within the broader political arena—gravitate toward Smart.

Wisdom is contemplative. Wisdom tends to accumulate over time and with life experience. Wisdom looks for subtleties, nuances, and is not fooled by seemingly simple solutions to complex problems. Wisdom is patient, knows that time is not static and is ever moving forward into a future that often brings clarity, understanding, and even resolution. When they can work together, Wisdom adores hanging out with Smart, knowing that they are an inspiring duo. But when Smart and Wisdom are not on the same page, Wisdom knows that nothing good can come of it.

## A Morality Tale

Smart and Wisdom walk into a bar. Smart orders a special brand of single malt whiskey, knowing it to be the perfect balance between quality and value. Since it is the middle of the day, and both must drive back to work, Wisdom settles for a diet coke. No sooner are they seated, when Smart decides to challenge Wisdom to a debate on whose defining characteristic is more important. Wisdom asks where this need to compete comes from and why such a debate is even necessary. Smart persists and begins to regale Wisdom on the virtues of being Smart. As Smart starts to talk louder, other bar patrons begin to take notice.

The crowd feels an immediate attraction to Smart, given it has shown up wearing an Armani suit and shiny black leather Hugo Boss shoes. Wisdom on the other hand is wearing a frumpy woollen cardigan, sweatpants, and Birkenstocks. Struggling to get a word in edgewise, Wisdom tries to impress on Smart that they work best together.

“Smart without wisdom is like Ying without Yang; like a fancy car without an engine; or like having a full tank of gas but nowhere to go.”

Smart does not really have a good come back, and so, suddenly jumps on top of one of the bar tables, shouting, “Smart can't be broken! Wise cracks!”

The room goes wild! Everybody loves it, although no one knows quite what it means. Wisdom can immediately sense the tide has turned, as Smart whips the crowd into a frenzy, while the room throbs to their chant, “Wise Cracks, Wise Cracks, Wise Cracks”! Wisdom slinks off into a corner, while Smart basks in the glow of victory and is plied with free drinks from patrons, raising numerous toasts to just how clever Smart has been.

## Epilogue

On the drive back to work in its BMW, Smart is in a motor vehicle accident in which two pedestrians are seriously injured. A blood alcohol test taken at the scene exceeds the legal limit. A court date is pending.

Wisdom stays on a while longer at the bar, largely ignored, sipping on what remains of its diet coke. Wisdom finally exits to no fanfare whatsoever, contemplating *what does all of this really mean and is there some bigger lesson to be learned?* Wisdom then drives its Volvo, always just ever so slightly below the speed limit, arriving back at work safe and sound.

## Conclusion

The inspiration to write this piece came in the context of observing and taking part in national discussions regarding medical assistance in dying. Like so many divisive issues, people seem to be more interested in racking up points than they are in listening, reflecting, and being open to opinions and evidence that might challenge their worldview. Being smart, shear intelligence, does not seem to confer immunity from this troubling inclination. Which is why, whether considering clinical work, setting health care policy, or deliberating broader political issues, I find myself compelled to write in praise of Wisdom.

Dr. Chochinov is a distinguished professor of psychiatry at the University of Manitoba and a senior scientist at CancerCare Manitoba Research Institute.

